# Factors Contributing to Maternal and Child Mortality Reductions in 146 Low- and Middle-Income Countries between 1990 and 2010

**DOI:** 10.1371/journal.pone.0144908

**Published:** 2016-01-19

**Authors:** David M. Bishai, Robert Cohen, Y. Natalia Alfonso, Taghreed Adam, Shyama Kuruvilla, Julian Schweitzer

**Affiliations:** 1 Department of Population, Family, and Reproductive Health, Johns Hopkins Bloomberg School of Public Health, Baltimore, Maryland, United States of America; 2 World Health Organization, Health Systems and Innovation, Geneva, Switzerland; 3 World Health Organization, Family, Women's and Children's Health, Geneva, Switzerland; 4 Results for Development, Washington, District of Columbia, United States of America; Université Catholique de Louvain, BELGIUM

## Abstract

**Introduction:**

From 1990–2010, worldwide child mortality declined by 43%, and maternal mortality declined by 40%. This paper compares two sources of progress: improvements in societal coverage of health determinants versus improvements in the impact of health determinants as a result of technical change.

**Methods:**

This paper decomposes the progress made by 146 low- and middle-income countries (LMICs) in lowering childhood and maternal mortality into one component due to better health determinants like literacy, income, and health coverage and a second component due to changes in the impact of these health determinants. Health determinants were selected from eight distinct health-impacting sectors. Health determinants were selected from eight distinct health-impacting sectors. Regression models are used to estimate impact size in 1990 and again in 2010. Changes in the levels of health determinants were measured using secondary data.

**Findings:**

The model shows that respectively 100% and 89% of the reductions in maternal and child mortality since 1990 were due to improvements in nationwide coverage of health determinants. The relative share of overall improvement attributable to any single determinant varies by country and by model specification. However, in aggregate, approximately 50% of the mortality reductions were due to improvements in the health sector, and the other 50% of the mortality reductions were due to gains outside the health sector.

**Conclusions:**

Overall, countries improved maternal and child health (MCH) from 1990 to 2010 mainly through improvements in the societal coverage of a broad array of health system, social, economic and environmental determinants of child health. These findings vindicate efforts by the global community to obtain such improvements, and align with the post-2015 development agenda that builds on the lessons from the MDGs and highlights the importance of promoting health and sustainable development in a more integrated manner across sectors.

## Introduction

Millennium Development Goal (MDG) 4 was to reduce child mortality by two thirds from 1990–2015 and MDG5 was to reduce maternal mortality by three quarters over the same time period. Child mortality has decreased by 49% and maternal mortality has decreased 45% worldwide since 1990, but the pace of reduction has varied across countries and 6.6 million children and 300,000 mothers continue to die every year from preventable causes [1–6]. The pace of mortality decline is not constant and its relationship to economic progress, political progress, and health system progress has varied over time and from place to place. Countries with similar geography, wealth, U5MR and MMR levels have shown wide differences in health progress over the last 40 years[7]. Several studies have been devoted to systematically accounting for past progress [8–11]. The direct relationship from maternal and child health (MCH) improvements to the alleviation of poverty has long been recognized [12]. Other key social determinants of health include better schooling [11], good governance [9], clean water [13], and less social inequality [14].

Health policy obviously involves scaling up of health sector based interventions, but health policy also involves policies to improve education, governance, the economy, the environment, and other social determinants of health. The impact of health interventions and social and environmental determinants (health determinants) on mortality has been known to change over time [15]. Preston’s seminal paper showed that changes in public health technologies between 1965 and 1975 made each dollar of national income growth a more important contributor to infant mortality over the decade. That particular decade of time passage from 1965 to 1975 was shown to have a particularly large effect on changing the β coefficient on GDP in determining child mortality. Obviously, it was not ten orbits around the sun that made economic growth more likely to lower mortality than before. Rather, this decade was marked by low income countries’ adoption of recent fruits of new scientific progress in sanitation, antibiotics, vaccines, and modern obstetrics. Public health progress in the 1960s made it possible to use new income in ways that were not present a decade earlier and made GDP a more impactful social determinant of health. Since Preston’s seminal paper, there has been little systematic study of whether the impact factors of macro health determinants is changing.

It is a good time to raise the question because the last 25 years of mortality declines have been paralleled by progress in coverage of most health determinants as well as progress in health technology (e.g. new treatments for AIDS, malaria, new vaccines, etc.). So it is difficult to know whether mortality declines are due primarily to higher coverage of health determinants or to improvements in the impact of health determinants. We focus on two co-contributing explanations for the decline in maternal and child mortality: 1) improvements in the absolute levels of key health determinants; 2) improvements in the impact of each health determinant since 1990, which includes secular progress due to time and diffusion of best practices. Health policies have focused on increasing the levels of social and intervention-based health determinants as well as getting more from each health determinant. Doing both is important. This paper asks how much of past improvements are due to higher levels of health determinants vs. improvements in the health impact per unit of change in a health determinant.

By examining country performance in MCH over the past twenty years we can assess some of the major factors responsible for the observed progress. A similar analysis was undertaken which found that maternal education, HIV, fertility, income and time accounted for 99% of the decline in child mortality [1]. Our study expands this analysis by examining a greater variety of health determinants and also assessing maternal mortality. Our goal in this paper is to assess how much of the world’s progress on MDG 4 and 5 has been due to increases in well-known determinants of MCH and how much progress has been due to changes in the impact of these determinants from 1990–2010. We decompose the magnitude of the observed improvement due to each of these possible explanations. The results can help policy-makers prioritize between strategies that make the levels of things like schooling, health spending, and good governance increase versus strategies that make schooling more likely to impact health, that make health spending more efficient, and that make governance improvements affect the health sector more positively.

## Methods

We examined the under-5 mortality rate (U5MR) and maternal mortality ratio (MMR) as the outcome variables since these indicators are used by the UN to monitor progress towards MDG 4 and 5. U5MR is the probability per 1,000 live births that a newborn baby will die before reaching age five if subjected to current age-specific mortality rates [16]. MMR is the probability that a mother will die within 42 days of her pregnancy per 100,000 live births. We completed our analysis on both the Inter-agency Group for Child Mortality Estimation (IGME) and the Institute for Health Metrics and Evaluation (IHME) Global Burden of Disease Study data [5,17]. The correlation between IHME and IGME U5MR values was 0.98 and the decomposition findings were very similar for both sources (see results). Results shown here are based on IGME’s estimates since these are the official United Nations estimates for measuring MDG 4.

Analysis included all countries classified as low- or middle-income in 2000 (GDP per capita > $9,266) with available U5MR, MMR, and per capita GDP data. This left 146 countries out of 155 eligible for analysis. We used multivariate regression analysis to decompose the decline in U5MR and MMR in these countries from 1990–2010. We classified over 250 different health determinants obtained from different public sources into eight different policy areas (Table B and Table C in S1 Appendix). Of these, we selected the 2–3 indicators from each policy area with empirically demonstrated relationship to MCH and data availability over 75%. The values for each health determinant were averaged over a five year interval (1988–1992 to generate an average estimate for 1990 and from 2008–2012 for 2010). Simple imputation was used to fill in missing data, and the analyses were run with raw data as well as with imputed data. Models were built using one variable from each of eight policy areas for U5MR and from six for MMR. Every possible combination of variables was chosen, leading to 384 unique models for U5MR and 216 for MMR. Since we ran all models using raw data and with imputed data, this led to 768 total models for U5MR and 432 for MMR. Given a sample size of 146, an attempt to stratify the analysis by world region would have been limited by low power and thus all 146 countries are aggregated.

The independent variables can be distinguished into two groups. More distal social determinants of health include wealth, environment, infrastructure and gender equality. Variables under the control of the health sector include: health service coverage, immunizations, and fertility. Including both social and health sector variables controls for confounding. Health service coverage is enhanced by infrastructure, economics, and education so it would be misleading to include one type of variable without the other.

Many studies have focused on providing a better theory to connect GDP growth to mortality growth in a way that explains the well-known non-linear relationship. Alternatives to a simple log log or log-linear model include an enzyme-kinetic model similar to the Michaelis-Menten relationship [18] and models including multiple lags of GDP [19]. An economic theory of mortality decline needs to recognize that pure money cannot change mortality, but that the money has to be used to purchase physical, social, and environmental changes that lower mortality. Simpler health production function models convey this by saying H = f(X)—that health, H, is a function, f of inputs, X. Appealing to standard functional forms used to model production processes like Cobb-Douglas or translog one could easily motivate a specification such as log(H) = C+βlog(X) +μ. In this model β can be interpreted as the production elasticity relating percentage improvements in input X to output H. In a one period model there would be a budget constraint stipulating that GDP must be used to finance these inputs as GDP = P_X_X + P_Z_Z where Z is all other goods in the economy and P_X_ and P_Z_ are the prices respectively of health determinants and all other goods. The budget constraint says that X and GDP will be correlated and one needs to be cautious about making any particular claims of causal attribution. Furthermore correlation between error term μ and X would create endogeneity bias because factors left out of the model might determine both H and X. This paper will show that the simple log log specification fits the data very well and then decomposes recent mortality declines into contributions from changes in β and contributions from changes in X. We are not focused on deriving an unbiased measurement of any particular β. Instead we want to know in general over thousands of estimates of β’s in hundreds of specifications whether the mortality improvements owe more to growth in β or growth in X.

### Procedures

Based on health production theory we claim that U5MR or MMR decline occurs as a result of A) Changes in the levels of health determinants symbolized by X; B) Changes in the impact of health determinants symbolized by β; and C) The interaction of changes in X with changes in β. A simple algebraic derivation of the standard regression equation can yield this framework known as the Oaxaca-Blinder (OB) decomposition as follows:

For any y=Xβ       Δy=βΔX+XΔB+ΔXΔβ (1)

We applied the OB decomposition to decompose the changes in U5MR between 1990 and 2010, into the sum of each of these three components [20,21]. The interaction term was usually small, and thus was not included in the comparison. The OB method works for linear models of outcomes that have a normal distribution, but the distribution of U5MR values is non-negative and skewed to have a long right tail. In order to apply the method the log of U5MR was used in all models. The analysis was done in Stata 12 using the add-in oaxaca.ado commands [22]. For details see Text A in S1 Appendix.

Endogeneity is an unavoidable concern because the error term for Δy—a mortality change—could directly correlate with Δ**X** which are the changes in health determinants. Endogeneity causes bias in the estimates of each separate β coefficient. However, our focus in this paper is not to present any specific β coefficient nor to make claims about which particular β coefficients are higher or lower than one another. What we want to know is the relative size of βΔ**X** vs **X**Δβ and in sensitivity analysis we cycle through nearly a thousand combinations of X variables to ensure that when we reach a conclusion that one of these two decomposition terms is bigger than another the conclusion does not depend on endogeneity bias that might distort the value of any particular β coefficient.

The regression models were run as weighted models. There are two plausible weighting schemes, and to avoid making an arbitrary choice we ran the data with weighting both by the number of births and then with weighting by the total population. Weighting each country by the number of births is plausible because one should weight by the number of people exposed to a mortality risk for both maternal and under 5 mortality. Others think that child or maternal mortality is a social concern that should scale with the number of people who are concerned rather than the number of people at risk of dying. In this view weighting by births under-values the lives of children in a large population country that has a low birth rate. Such a country would have a lower total number of death events, but a higher total number of citizens who are concerned about these events and what they signify for the quality of life of their country. To avoid resting all results on an arbitrary choice of weights we ran all models with both weighting schemes as a sensitivity check. It turned out that both birth-weighted and population-weighted models reached the same conclusion regarding the overall decomposition.

After completing the main decomposition we sought to determine whether governance and per capita GDP growth were confounded with improvements in other determinants of health. We regressed the absolute increase in each health determinant’s level as the dependent variable and four independent variables: average annual GDP per capita growth since 1990, starting GDP per capita in 1990 (or the earliest year available), improvement in the World Governance Indicators government effectiveness index from 1996–2010 (the only available years), and starting government effectiveness index in 1996. We ran these regressions using all countries in the dataset. This check on the role of GDP and governance in confounding other health determinants was implemented as follows:

$$\Delta \text{x}=\beta_{0}\;+\;\beta_{0}\text{Log}\left({\text{GDPpc}}_{1990}\right)+\;\beta_{0}\left(\text{Gov}.{\text{ Effectiveness Index}}_{1996}\right)\;+\;\beta_{0}\Delta \text{Log}\left(\text{GDPpc}\right)\;+\;\beta_{0}\left(\Delta \text{Gov}.\text{ Eff}.\right)\;+\;\varepsilon_{\text{j}}$$ (2)

The R^2^ in the model (2) tells us how much of the variance in Δx was explained by governance and economic growth.

### Sensitivity Analysis

In sensitivity analysis alternative models were estimated that did not transform U5MR or MMR, but performed OB decomposition based on zero truncated Poisson models using nldecompose.ado (Table D in S1 Appendix). [22]. The analysis uses population weighting, but we also examined the results from weighting by the number of births. Both weighting schemes generate roughly similar conclusions.

## Results

There were 146 countries with year 2000 GDP per capita < $9,266 and reported values of U5MR and MMR in the analysis. The population weighted average U5MR for this group of countries was 87 (Min 7-Max 326) in 1990 and 44 (Min 4-Max 193) in 2010. Thus there was a population-weighted drop of 43 deaths per 1000 in U5MR between 1990 and 2010. The population weighted average MMR was 333 (Min 8-Max 2300) in 1990 and 175 (Min 2-Max 1200) in 2010, for a population-weighted drop of 158 deaths per 100,000 live births over the 20 years.

**Fig 1:**
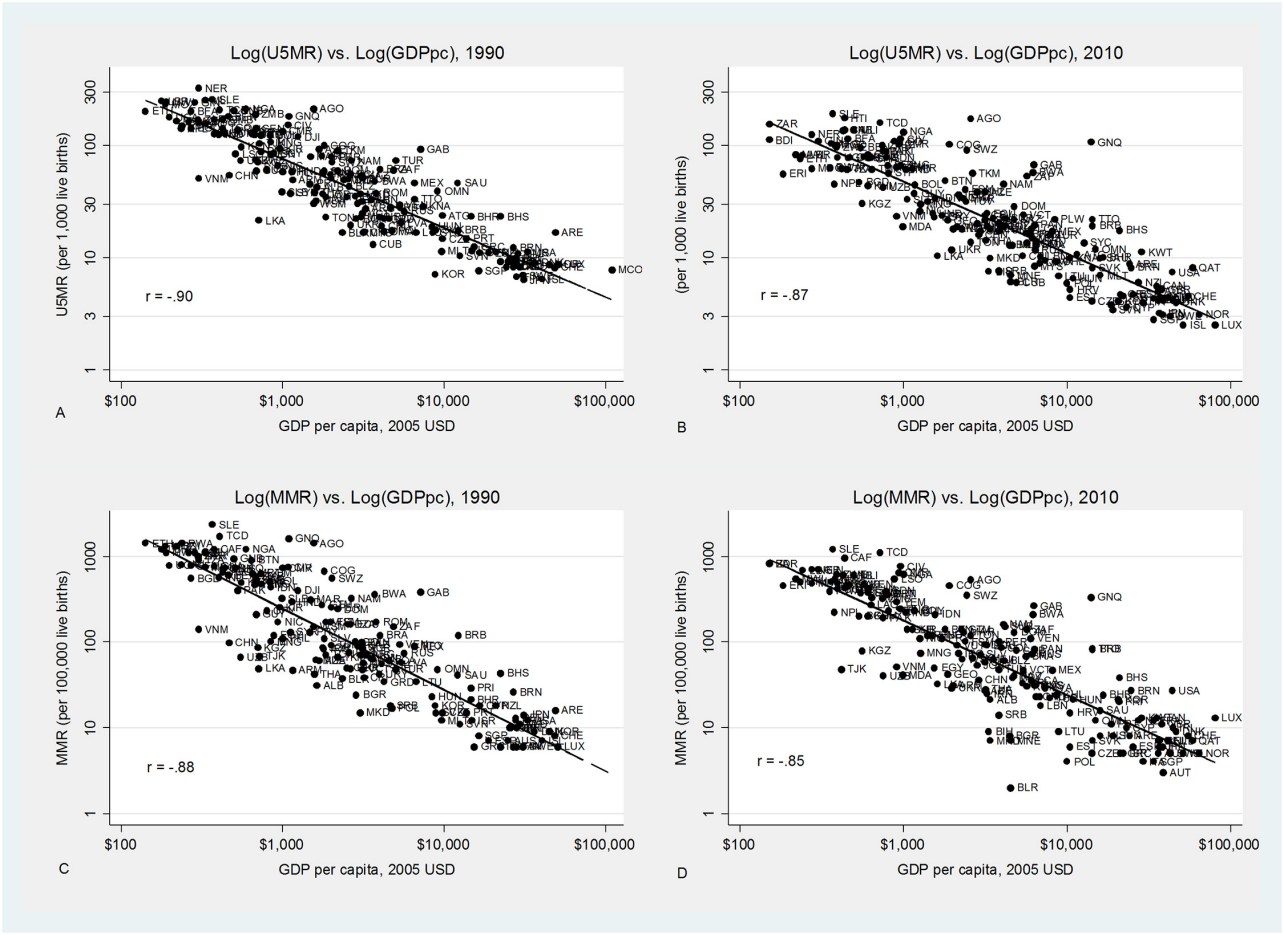
Relationship between both Log (U5MR) and Log (MMR) and Log (GDP per capita). The well-known Preston relationship is clearly visible, with correlation coefficients of between -.8 and -.9. A line of best fit curve is added showing a similar slope for both years, as well as a shift rightward and downward in 2010 compared to 1990. There are 170, 179, 162, 173 countries shown respectively (A-D).

Fig 1 shows a close relationship between Log(GDP per capita) and both Log(U5MR) and Log(MMR). The slope of the curve stayed roughly the same but the entire curve shifted downward and rightward from 1990–2010. The rightward shift reflects the effects of increasing world income with its corresponding effect on health, while the downward shift indicates that existing resources, including but not limited to income, had a stronger impact on health in 2010. However, there is also considerable variance among countries with similar per capita GDP, which must be explained.

The social factors associated with reductions in child mortality showed substantial improvements from 1990 to 2010 (Table 1). LMICs in aggregate improved nationwide coverage for every health determinant included in the models. Indeed, the levels of a wide variety of known child health determinants have improved significantly since 1990, and have increased faster in countries which have shown greater child health improvements (Figure A in S1 Appendix).

**Table 1:** Summary statistics for sample of countries showing change in U5MR and change in factors responsible for U5MR. Observations are available raw data. Means listed include imputed values.

		1990				2010				Abs. Change
Policy Area	Variable	Obs	Mean	Min	Max	Obs	Mean	Min	Max	1990–2010
**Non-Health Sector**										
Wealth	Log GDP per capita, 2005 USD	134	6.7	4.9	9.6	146	7.5	5	9.9	**0.8**
	Log GDP pc PPP, 2011 international $	134	8	6	10.5	145	8.8	6	10.8	**0.8**
Environment	Log odds Clean Water Access	124	1.1	-1.9	5.3	146	2.2	-0.5	5.3	**1.1**
	Log odds Improved Sanitation Access	115	-0.5	-4	5.3	146	0.5	-2.4	5.3	**1**
Infrastructure	Rural Electricity Access	146	62.2	0.1	100	146	74.9	0.3	100	**12.7**
	Urbanization %	146	36.6	5.7	88.9	146	46.6	10.7	93.3	**10**
	Log Road Density, km roads per sq. km	96	-1.6	-5.4	1.4	146	-1.1	-4.6	1.7	**0.5**
Education	Lag 10 years Gross Female Primary School Enrollment %	115	88.1	13.2	197	146	94.4	23.3	141	**6.4**
	Lag 5 years Gross Female Secondary School Enrollment %	99	35.1	-1.2	107	146	59.1	7.6	116	**23.9**
	Lag 5 years Gross M&F Secondary School Enrollment %	120	41.6	3.4	111	146	60.9	9.9	116	**19.3**
Gender Equality	Female Labor Force Participation %	136	37.1	10.6	55.7	146	37.4	14.2	53.4	**0.4**
	Parliamentarians in National Legislatures % Women	99	11.5	-3.7	36.9	146	16.6	0	56.3	**5.1**
**Health Sector Factors**										
Health Service Coverage	Log odds Skilled Birth Attendance %	72	0.8	-2.6	5.3	146	2.1	-2.3	6.4	**1.3**
	Log Physicians per 1,000 population	131	-0.5	-5	1.8	146	-0.3	-4.8	1.9	**0.2**
	Log odds Prenatal Care	40	5.4	2.9	9.9	146	6.8	4.3	10.3	**1.4**
Immunizations	Log odds Measles Immunization, % one-year-olds	140	1.2	-5.3	5.3	146	2.7	-0.2	5.3	**1.5**
	Log odds DPT Immunization, % one-year-olds	140	1.3	-5.3	5.3	146	2.7	-0.7	5.3	**1.3**
Fertility	Log Total Fertility Rate (Births per woman)	144	1.2	0.5	2.2	146	0.9	0.2	2	**-0.3**
	Log Adolescent Fertility Rate (Births per 1,000 women age 15–19)	141	3.9	1.6	5.4	146	3.5	0.8	5.3	**-0.5**
	Log Lag 5 years TFR (Births per woman)	143	1.3	0.5	2.2	146	0.9	0.1	2	**-0.4**

**Fig 2:**
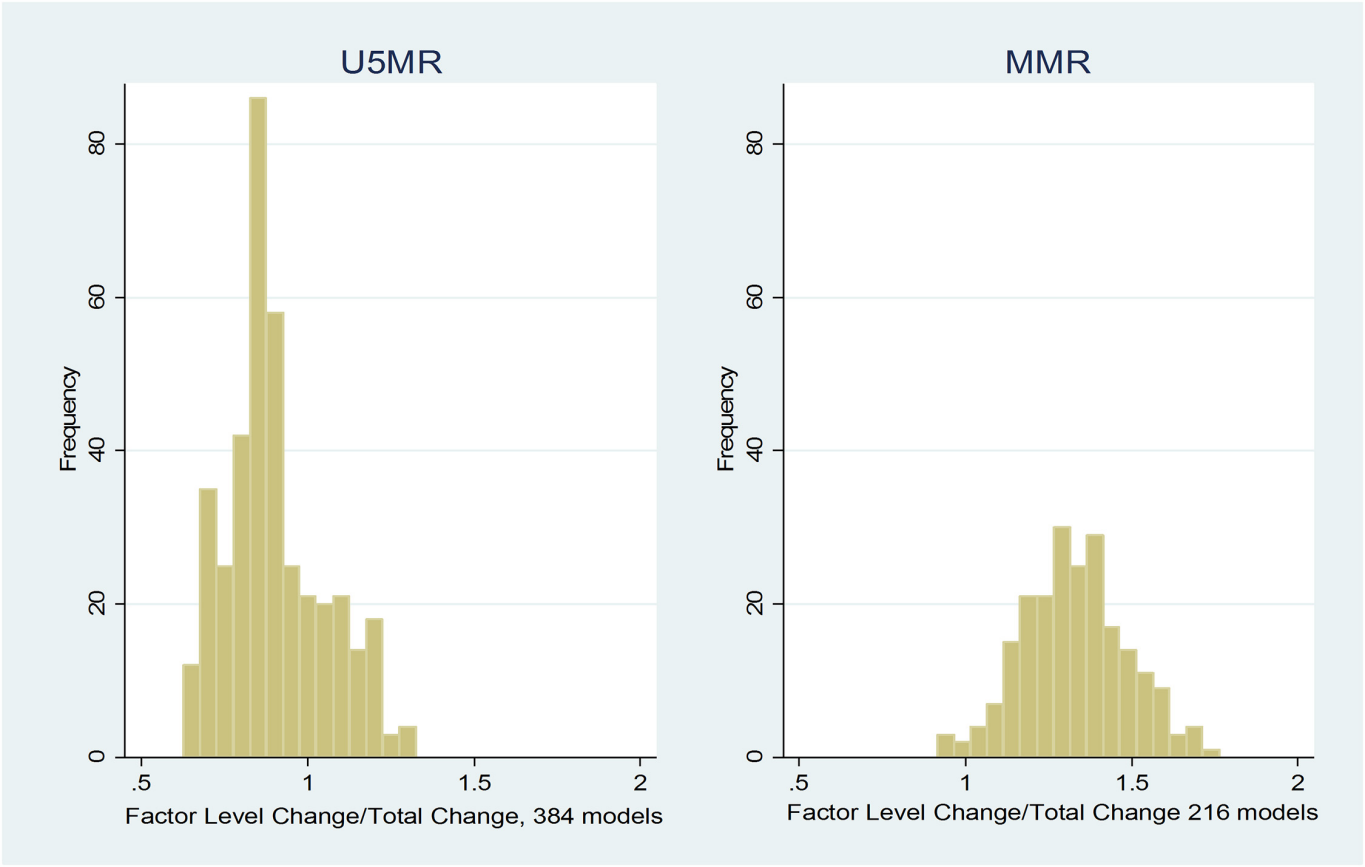
Histograms. Value examined is the relationship of changes in societal coverage level compared to total accounted changes, or (βΔ**X**/(βΔ**X** + **X**Δβ). The interaction term Δ**X**Δβ was small, usually negative, and thus ignored (Table A in S1 Appendix).

Fig 2 shows that across all 768 models for U5MR, the average contribution of improved societal coverage of health determinants was 89%, with an average of 87% (Interquartile range (IQR): 76%-99%) for 384 models without imputed data and 90% (IQR: 80%-99%) for 384 models with imputed data. In other words, 89% of the improvement in children saved between 2010 and 1990 is attributable to gains in health determinants across the eight policy areas tested in our models. For MMR, the pattern is similar, with one insignificant difference. The overall mean contribution of improved societal coverage of health determinants across 432 models was 141%, with an average of 149% (IQR: 99%-187%) for 216 models without imputed data and an average of 133% (IQR: 122%-143%) for 216 models with imputed data. Improved societal coverage is represented as βΔ**X** and can be greater than 100% if β and/or Δ**X**Δβ is less than 100% and this is the case for MMR. Many of the β coefficients relating social determinants to MMR got smaller between 1990 and 2010 and Δβ overall was negative. For MMR, one must attribute all of the improvement to changes in social determinants and not to improvements in the impact factor of these determinants.

**Fig 3:**
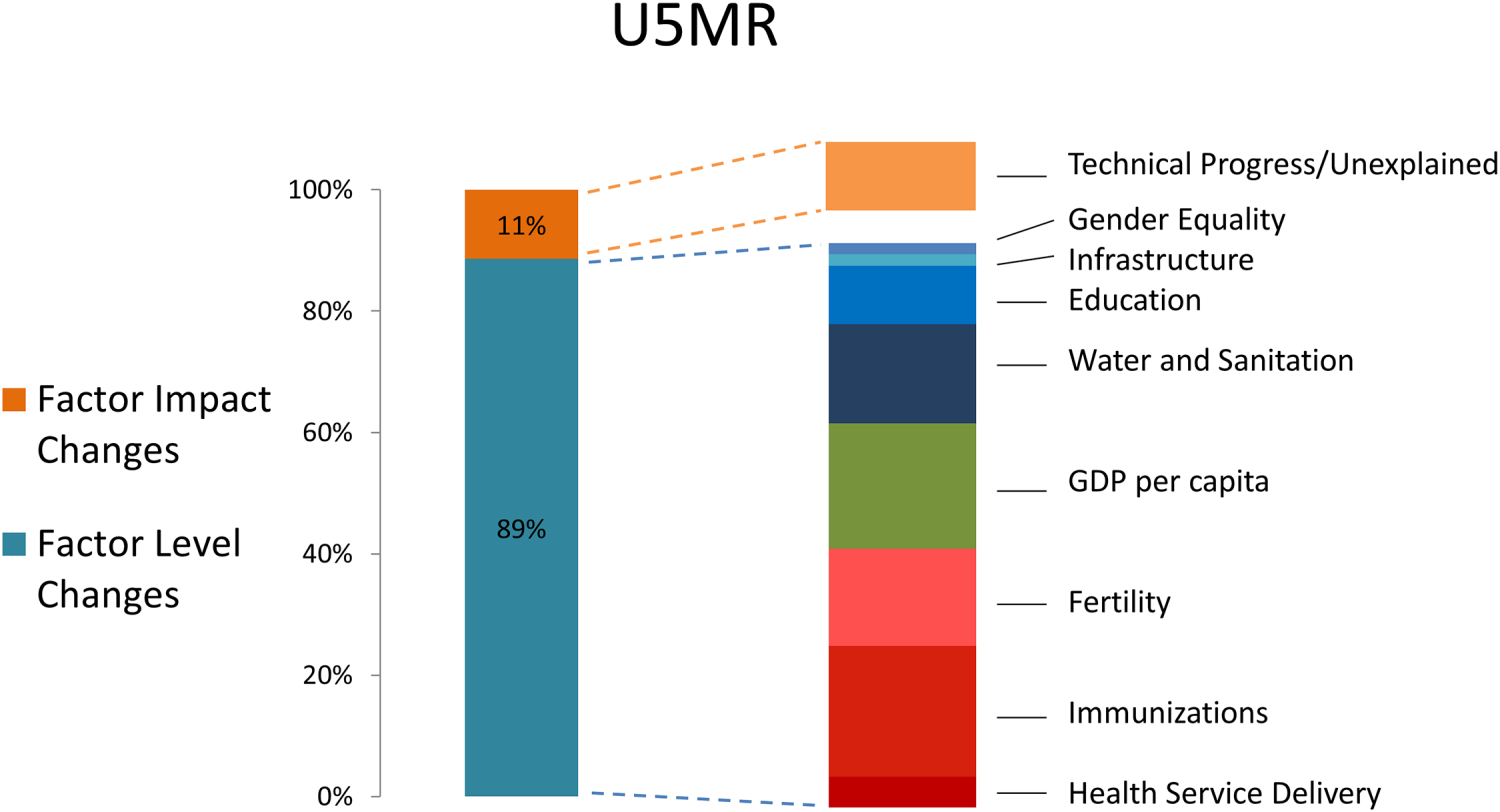
Contribution of changes in the levels of determinates of health (health interventions, social and environmental determinants) to reductions in U5MR, 1990–2010 (A). Out of 768 models run, the contribution of factor level changes averaged 89%. Of these, half included a variable for fertility (Log of TFR lagged 5 years), and half did not out of concern for endogeneity. The decomposition to the right shows how these improvements averaged a breakdown by indicator. Details of this latter composition are shown in Table A in S1 Appendix. For uncertainty ranges around each estimate see Figure B in S1 Appendix.

**Fig 4:**
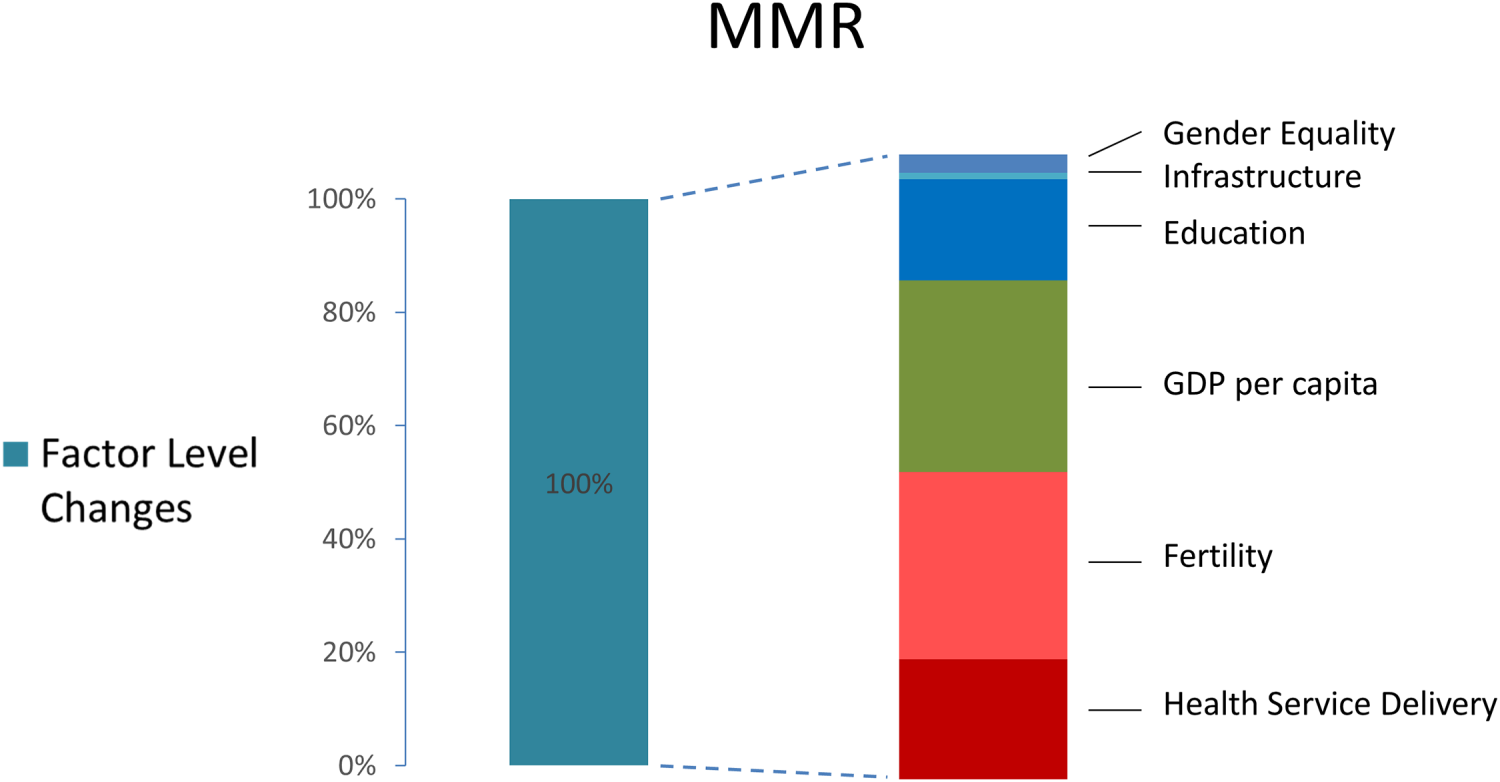
Contribution of changes in the levels of determinates of health (health interventions, social and environmental determinants) to reductions in MMR, 1990–2010. Out of 432 models run, the contribution of factor level changes averaged 133%, which is rescaled to 100% here. The decomposition to the right shows how these improvements averaged a breakdown by indicator. For uncertainty ranges around each estimate in the see Figure B in S1 Appendix.

Fig 3 shows the mean contribution of each sector to LMIC deaths averted for U5MR, and Fig 4 shows the corresponding contributions for MMR. These figures must be interpreted with caution. Changing the variables in each model shifts the percent contribution for each sector somewhat. Subject to these limitations, the growth in GDP per capita accounts for just 20% (IQR: 15–26%) of total improvements in child health since 1990. GDP per capita increases accounts for 31% (IQR: 23–37%) of MMR decline. Detailed results for the average contribution from various policy areas are shown in Figure B in S1 Appendix. Increased immunizations, improved water and sanitation, and secondary schooling all contributed significantly to child health. Reductions in fertility and increases in secondary schooling and skilled birth attendance all contributed approximately one quarter of gains in maternal health. The range of percent contributions from each policy area varied, but in nearly all models the change in the level of health determinants (ΔX) accounted for the majority of the gains compared to changes in the coefficients (Δβ). Furthermore, consistently across models, approximately 50% of the mortality reductions were due to improvements in the coverage of variables representing the health sector, and the other 50% were due to improvements in those variables outside the health sector.

Each variable was highly correlated with other variables from the same policy area. For example, replacing measles immunization with immunization against DPT3, Polio, or BCG would result in a similar contribution. It would be inappropriate to conclude that it was specifically these particular variables, and not others, that contributed to mortality declines. Advances in broadly correlated processes that direct social function towards better well-being are the more likely the determinant of mortality decline.

We checked whether the gains in social and technical determinants of health were just proxies for improved governance and GDP growth. This is a substantial threat because U5MR and MMR estimates are reliant on models that might include GDP. Multivariate regressions of health determinant improvements against governance and growth show that economic growth and governance accounted for only 2–20% of the observed level of factor improvements, and usually less than 10% for the most impactful factors (Table 2). However, the heterogeneous nature of the study population and the fact that many of the variables used in the data represent average values over a number of years could also partly explain the observed low R2 values.

**Table 2:** Amount of improvements accounted for by just economic growth and governance. The absolute change in each factor in the table was the dependent variable, and the independent variables were annual per capita GDP growth from 1990–2010, improvement in governance 1996–2010, starting GDP per capita in 1990, and starting government effectiveness in 1996. When data were not available for the given year, the most recent available data was substituted. The percent explained is the R^2^ term from unweighted OLS regression.

Variable	Percent of variance explained by economic growth and governance (R^2^)
Non-Health Sector Factors	
Log odds clean water access	14.30%
Log odds sanitation access	14.20%
Lag 5 years secondary school M&F enrollment	12.60%
Log road density	10.70%
Lag 5 years secondary school female enrollment	9.40%
Female labor force participation	8.80%
Rural electricity access	5.90%
National Parliamentarians, % Women	5.90%
Lag 10 yrs. gross primary school female enrollment	5.50%
Urbanization	5.00%
Health Sector Factors	
Log U5MR	16.50%
Log MMR	18.90%
Log adolescent fertility rate	19.20%
Log lag 5 yrs TFR	13.40%
Log TFR	7.90%
Log odds skilled birth attendance	7.20%
Log odds DPT vaccination	6.90%
Log odds measles vaccination	5.70%
Log physicians per 1,000	4.40%
Log odds prenatal care	2.50%

## Discussion

In the period after World War II, low and middle income countries had the luxury of facing a world of technical progress. Mortality rates could fall substantially without the need for economic growth because the impact factor of GDP on mortality was dramatically improving thanks to new discoveries in public health. Just riding on technology could save more of the people who were already covered by the modern social system without the necessity and expense of expanding the umbrella. The twenty years between 1990 and 2010 brought even more wonderful new discoveries in treatments for HIV, new vaccines, and new innovations in sanitation and health communication, but there has also been basic progress in expanding the umbrella of coverage in this period. This paper has measured the relative contributions of expanding basic factors that lead to health vs. progress in making those factors more impactful. There is a decisive answer. Improvement in the levels of health determinants explains nearly all of the progress on U5MR and MMR. For MMR the impact factors for social determinants have gotten smaller between 1990 and 2010. For U5MR, impact factor improvement accounts for only 11% of the reductions between 1990 and 2010.

No single health input dominated as the cause of mortality decline. Rather, the summation of improvements in many different policy areas is what saved lives. Economic growth and good governance, while valuable ends in themselves, were only part of a broad area of health determinants whose progress improved child health. Improvements in factors from diverse sectors as the environment, education, the health system, fertility, and women’s empowerment all contributed significantly to mortality reductions. These results point to the importance of a multi-sector approach to improving child health and thus aligns with the findings of country case studies, qualitative analysis, and literature review from the larger MDGs 4 and 5 Success Factors Study [23]. They also suggest that the eight policy areas included in our model—the same ones emphasized by successful LMICs and development partners—are those of great importance to MCH. Our findings vindicate efforts to address a multisectoral set of key health determinants as a path to reach the health MDGs.

We considered whether what we interpreted as “multi-sectoral” progress was really just a proxy for economic growth and good or improved governance driving progress across all other sectors. We find that these two factors by themselves were only minor predictors of either gains in child health or in the other factor levels that drove child health. As a second test of this finding, the OB decomposition similarly showed that economic growth accounted for an average of 20% of child health gains and 31% of maternal health gains across all models tested.

All models are subject to limitations. Concerns about endogeneity and unobservable confounders are unavoidable in cross-country regressions. There are limitations related to the errors induced in the way MMR and U5MR have been constructed based on models. However, we attempt to mitigate these concerns by testing over 1000 models, including both unimputed data and imputed data. The findings for each policy area were similar whether missing data was filled in by imputation or not, with the largest differences noted for highly imputed variables. Many cross-country regressions are of dubious value because their results hinge on model specification—on the particular combination of included variables. Our findings were robust across 600 combinations of health determinants.

Still, the findings should be interpreted with care. Variables within and even between policy areas are highly correlated, and interchanging one for another usually did not alter significantly the contribution of that policy area. Thus, readers should not examine the results to find support for any single particular factor as being able to deliver health gains. We refrain from drawing specific conclusions about the causal significance of individual coefficients on individual health determinants. It is also possible that the contribution of different health determinants to MCH varies by region, and we did not test for that here because the sample size is only 146 countries.

The main conclusions that can be drawn from this analysis are that 1) it is the improved levels more so than improvements in the impact per unit of a combination of proven health determinants that contributed to declining mortality, 2) mortality decline was not solely due to GDP growth, but was independently lifted by multisectoral progress. This analysis should not be taken to discount the importance of improvements in specific health conditions. Our analytical approach used U5MR and MMR as summary indicators of health. Progress on these specific health conditions obviously matters substantially for U5MR and MMR, but decomposing that progress would require a separate analysis that is out of our scope.

Looking ahead to a post-2015 agenda for Sustainable Development Goals these results indicate that progress on the health of mothers and children will depend on multiple underlying determinants improving at the same time. Countries and donors must invest in a multi-faceted approach across multiple policy areas to improve MCH. Countries cannot count on technical progress to make existing levels of health determinants more impactful on human health. A broad array of both health sector interventions and social determinants of health need to improve together. The post-2015 development agenda with an emerging emphasis on multi-sector collaboration offers an unprecedented opportunity to build on the lessons from the MDGs and to promote health and sustainable development in a more integrated manner.

## Supporting Information

S1 Appendix Supporting Information. Addition details about the methods (Methods A). Sensitivity analysis to show how results change for birth weighting vs. population weighting (Table A) and for imputation vs. no imputation (Tables B and C) and to models using zero-inflated Poisson models (Table D). Additional detailed results about how much each variable changed between 1990 and 2010 (Figures A and B). Stata code used in the analysis (Text A).
(DOCX)
