# Supplementary material for: Factors Contributing to Maternal and Child Mortality Reductions in 146 Low- and Middle-Income Countries between 1990 and 2010
Source: PLoS One. 2016 Jan 19;11(1):e0144908. doi: 10.1371/journal.pone.0144908 (PMC4718632; doi:10.1371/journal.pone.0144908)
Supplement: S1 Appendix — Addition details about the methods (Methods A). Sensitivity analysis to show how results change for birth weighting vs. population weighting (Table A) and for imputation vs. no imputation (Tables B and C) and to models using zero-inflated Poisson models (Table D). Additional detailed results about how much each variable changed between 1990 and 2010 (Figures A and B). Stata code used in the analysis (Text A). (DOCX) [file pone.0144908.s001.docx]

**Factors contributing to child mortality reductions in 146 low- and middle-income countries between 1990 and 2010**

APPENDIX CONTENTS

[METHODS 1](#_Toc439262130)

[SENSITIVITY ANALYSIS 3](#_Toc439262131)

[RESULTS 7](#_Toc439262132)

[STATA CODE 9](#_Toc439262133)

[BIBLIOGRAPHY 10](#_Toc439262134)

# METHODS

**Data Sources**

Data for outcome and independent variables of interest were extracted and compared between the following sources: Institute for Health Metrics and Evaluation (IHME),[[1](#_ENREF_1)] WHO Global Health Observatory [[2](#_ENREF_2)], WHO National Health Accounts (NHA),[[2](#_ENREF_2)] UN data,[[3](#_ENREF_3)] UNDP,[[4](#_ENREF_4)] UNICEF’s Childinfo,[[5](#_ENREF_5)] the World Bank DataBank,[[6](#_ENREF_6)] and Demographic and Health Surveys (DHS).[[7](#_ENREF_7)]

| Source | URL | Citation |
| --- | --- | --- |
| Institute for Health Metrics and Evaluation (IHME) | <http://ghdx.healthdata.org/ihme_data> | [[1](#_ENREF_1)] |
| WHO Global Health Observatory | <http://www.who.int/gho/database/en/> | [[2](#_ENREF_2)] |
| WHO National Health Accounts (NHA) | <http://apps.who.int/nha/database> | [[2](#_ENREF_2)] |
| UN data | <http://data.un.org/> | [[3](#_ENREF_3)] |
| UNDP | <http://hdr.undp.org/en/data> | [[4](#_ENREF_4)] |
| UNICEF’s Childinfo | <http://data.unicef.org/> | [[5](#_ENREF_5)] |
| World Bank DataBank | <http://data.worldbank.org/> | [[6](#_ENREF_6)] |
| Demographic and Health Surveys (DHS) | <http://dhsprogram.com/data/> | [[7](#_ENREF_7)] |

**Time periods**

We examined the decline in U5MR and MMR from 1990-2010.

**Selection of countries**

Analysis was confined to the 193 UN member states plus the Occupied Palestinian Territories. Countries were excluded from each analysis if they had no reported values for GDP or the outcome variable in question from 1990-2010. We also excluded all countries classified as high-income by their 2000 GDP per capita (defined as $9,266 [[8](#_ENREF_8)]  in 2000 US dollars) from the analysis. These criteria left 146 countries eligible for the analysis. Countries were grouped in sub-regions as defined by the UN statistics division.[[9](#_ENREF_9)]

**Dependent variable and problems with modeled data**

U5MR estimates from the Interagency Group for Child Mortality Estimation (IGME) [[10](#_ENREF_10)] were used. These are based primarily on the results from household surveys, as opposed to child mortality estimates from the Institute for Health Metrics and Evaluation (IHME) [11], which are based on both surveys and models including GDP, maternal education, and HIV. The IGME data were selected for three main reasons: 1) these data are used as the benchmark for MDG4; 2) they do not use covariates in mortality estimation, which would cause significant confounding in our study, 3) The correlation between IGME and IHME data was 0.98. Running the analyses with either choice of dependent variable source did not change the findings.

Maternal Mortality estimates were used from the most comprehensive time series available [12]. These data, which are based on modeled estimates, have benefits as well as drawbacks. It is the only available dataset that would permit a global analysis. The fact that it is based partly on modeled data is a limitation to be accounted for in interpretation of the results.

**Independent Variables**

Since maternal and child mortality are multifactorial and at least somewhat related to economic development, a broad perspective on health and societal development was used to select independent variables to include in models. Firstly, the UN suggests that plans designed to meet the MDGs should include investments in seven “clusters” of public policy.[[13](#_ENREF_11)] These are, in brief: 1) rural infrastructure, 2) urban improvement, 3) a well-functioning health system, 4) universal primary education, 5) gender and socioeconomic equality, 6) environmental management and access to water, and 7) economic growth. We further considered a health system to be comprised of six building blocks as defined by the World Health Organization (WHO)[[14](#_ENREF_12)]: 1) service delivery, 2) human resources, 3) information systems, 4) financing, 5) technologies, and 6) governance. We considered indicators for each of these priority areas from a list of over 250 related indicators compiled by a team of World Bank and WHO experts on maternal and child health in a previous study[[13](#_ENREF_13)] which also met these additional criteria: i) they fit into any one of the above policy or health systems priority areas ii) showed significant statistical correlation with reductions in maternal and child mortality iii) the statistical relationship was supported by the empirical and theoretical literature iv) data was at least 75% complete from 1990-2010. We made an exception for skilled birth attendance and prenatal care, since these determinants have strong support in the literature and were the best available for the important policy area of health systems.

# SENSITIVITY ANALYSIS

**Sensitivity analysis by weighting scheme**

There is a trivial difference in output between population-weighted and birth-weighted 1990-2010 baseline models. The main analysis uses population weighting. A country with a large population would have many more people to experience concern about the levels of U5MR even if it had only a few births. Population weighting gives more weight to health problems in proportion to the national population concerned about it. An alternative weighting scheme was also used in which U5MR was weighted by the number of births under an epidemiological perspective that births are at risk and the weight should be proportional to risk exposure. It turns out that both weighting schemes generate roughly similar conclusions (Table D). This model uses the default value for each policy area: GDP per capita, clean water access, skilled birth attendance, lag 10 years girls primary school attendance, lag 5 years total fertility rate, measles immunization, rural electricity access, and % of parliamentarians that are women.

Appendix Table A Sensitivity analysis of two weighting schemes. Table shows endowments and coefficients emerging from Oaxaca decomposition. Results at left apply weights proportional to total country population. Results at right apply weights proportional to birth count.

|  | Population Weighted | | | | | | | | Births Weighted | | | | | | | |
| --- | --- | --- | --- | --- | --- | --- | --- | --- | --- | --- | --- | --- | --- | --- | --- | --- |
| VARIABLES | Differential | | Endowments | | Coefficients | | Interaction | | Differential | | Endowments | | Coefficients | | Interaction | |
|  |  | |  | |  | |  | |  | |  | |  | |  | |
| Log GDP per capita |  | | 0.12 | | 0.553 | | -0.057 | |  | | 0.078 | | 0.319 | | -0.028 | |
| Log odds Clean Water Access |  | | 0.099 | | 0.118 | | -0.06 | |  | | 0.067 | | 0.021 | | -0.011 | |
| Log odds Skilled Birth Attendance |  | | 0.136 | | 0.08 | | -0.049 | |  | | 0.082 | | 0.029 | | -0.022 | |
| Lag 10 years Primary School Attendance, Female |  | | 0.006 | | 0.194 | | -0.012 | |  | | 0.008 | | 0.167 | | -0.011 | |
| Lag 5 years Total Fertility rate |  | | 0.23 | | 0.068 | | 0.029 | |  | | 0.183 | | 0.042 | | 0.013 | |
| Log odds Measles Immunization |  | | 0.155 | | -0.018 | | 0.01 | |  | | 0.169 | | 0.082 | | -0.047 | |
| Rural Electricity Access |  | | -0.01 | | -0.272 | | 0.046 | |  | | 0.007 | | -0.175 | | 0.026 | |
| Parliamentarians and Legislators, % women |  | | 0.017 | | 0.212 | | -0.065 | |  | | 0.025 | | 0.143 | | -0.052 | |
| CUTOFF |  | | -0.012 | | 0.372 | | -0.003 | |  | | -0.001 | | 0.386 | | 0 | |
| Total |  | | 0.741 | | 0.219 | | -0.163 | |  | | 0.618 | | 0.185 | | -0.132 | |
| Differential | . | |  | |  | |  | | . | |  | |  | |  | |
| Prediction_1 | 4.262 | |  | |  | |  | | 4.432 | |  | |  | |  | |
| Prediction_2 | 3.466 | |  | |  | |  | | 3.761 | |  | |  | |  | |
| Difference | 0.797 | |  | |  | |  | | 0.67 | |  | |  | |  | |
| Constant |  | |  | | -1.089 | |  | |  | |  | | -0.829 | |  | |
| Observations | 283 | | 283 | | 283 | | 283 | | 275 | | 275 | | 275 | | 275 | |
| Percent Due to Endowments |  | 77% | |  | |  | |  | | 77% | |  | |  | |  |

**Sensitivity analysis to Imputation by Model**

This table shows how much the decision to impute missing variables had on the effect of various indicators. The results in the final two columns display the percent of the change in U5MR accounted for by each changes over time in each indicator based on the average of between 64 and 96 various models using the indicator. For Log GDP per capita imputing lowers the importance from 19.8 to 18.4%. Schooling measures were those most sensitive to the decision to impute.

Appendix Table B Sensitivity to imputation for U5MR models

| **Policy Area** | **Indicator** | **Number of models** | **Models with no imputation*** | **Models with imputation*** |
| --- | --- | --- | --- | --- |
| GDP per capita | Log GDP per capita | 96 | 19.8% | 18.4% |
| Health Service Delivery | Skilled Birth Attendance | 96 | 4.3% | 8.6% |
| Human Resources for Health | Doctors per 1,000 | 96 | -3.8% | -3.0% |
| Immunizations | Measles Coverage | 96 | 16.6% | 20.0% |
|  | DPT Coverage | 96 | 12.1% | 16.3% |
| Fertility | Log Lag 5 years TFR | 96 | 31.8% | 34.3% |
| Water/Sanitation | Clean Water, % Access | 96 | 17.1% | 15.3% |
|  | Sanitation, % Access | 96 | 21.2% | 18.0% |
| Education | Lag 10 girls primary enrollment | 64 | -0.8% | 1.5% |
|  | Lag 5 girls secondary enrollment | 64 | 19.7% | 8.8% |
|  | Lag 5 gross secondary enrollment | 64 | 16.7% | 7.0% |
| Infrastructure | Rural Electricity, % Access | 96 | -0.5% | -0.8% |
| Urbanization | % Urbanized | 96 | 7.2% | 4.9% |
| Gender Equality | Legislators, % Women | 96 | 2.0% | 3.5% |
|  | Labor Force Participation, Women | 96 | -2.6% | 0.3% |

Appendix Table C Sensitivity to imputation for MMR models

| **Policy Area** | **Indicator** | **Number of models** | **Models with no imputation*** | **Models with imputation*** |
| --- | --- | --- | --- | --- |
| GDP per capita | Log GDP per capita | 108 | 41.8% | 30.6% |
| Health Service Delivery | Skilled Birth Attendance | 72 | 33.0% | 36.0% |
|  | Prenatal Care, % | 72 | 12.3% | 18.2% |
| Human Resources for Health | Doctors per 1,000 | 72 | 8.6% | 3.2% |
| Fertility | Log TFR | 108 | 45.3% | 41.8% |
|  | Log adolescent fertility rate | 108 | 16.8% | 18.2% |
| Education | Lag 10 girls primary enrollment | 172 | -1.8% | -3.0% |
|  | Lag 5 girls secondary enrollment | 72 | 19.9% | 25.8% |
|  | Lag 5 gross secondary enrollment | 72 | 21.1% | 25.8% |
| Infrastructure | Rural Electricity, % Access | 72 | 4.3% | 13.5% |
|  | Log road density, % sq. km | 72 | -13.0% | -3.6% |
| Urbanization | % Urbanized | 72 | 1.2% | -6.5% |
| Gender Equality | Legislators, % Women | 108 | 2.2% | 5.5% |
|  | Labor Force Participation, Fem. | 108 | 0.0% | 0.3% |

Se**nsitivity analysis by regression model**

To see if the results in the main text were due to the use of a linear model, we also explored the use of the Poisson model of under 5 mortality . This model used the default indicator variable for each policy area: GDP per capita, clean water access, skilled birth attendance, lag 10 years girls primary school attendance, log odds measles immunization, lag 5 years total fertility rate, rural electricity access, and % of parliamentarians that are women. There are three possible assumptions about the weighting matrix. Omega=1 derives beta exclusively from the 2010 dataset. Omega=0 derives the coefficient exclusively from the 2010 data and. Omega=Neumark is a weighted average of coefficients from both 1990 and 2010 using a weight matrix. Results from decomposing using the zero inflated Poisson regression of untransformed U5MR showed that the change in health determinants accounted for 85.3% of the gap and factor impact accounted for 14.6% of the gap, which is similar to the result found in the main analysis.

**Table D. Sensitivity to Use of Zero Inflated Poisson Regression**

Number of obs (A) = 142

Number of obs (B) = 133

----------------------------------------------------------------------

Results | Coef. Percentage

--------------+-------------------------------------------------------

Omega = 1 |

Char | -39768.18 96.74137%

Coef | -1339.55 3.258633%

--------------+-------------------------------------------------------

Omega = 0 |

Char | -33883.02 82.42494%

Coef | -7224.709 17.57506%

----------------+-----------------------------------------------------

Omega = Neumark|

Prod | -35096.04 **85.37578%**

Adv | -3717.014 9.04213%

Disadv | -2294.67 5.582088%

--------------+-------------------------------------------------------

Raw | -41107.73 100%

----------------------------------------------------------------------

# RESULTS

**Absolute difference in factor levels from 1990-2010 for 23 child health determinants**

When data was missing for 1990 or 2010, the closest available data entry in time was substituted. Means are not weighted by population in order to prevent domination of the analysis by China and India. The 10 Success Factor countries have been identified as the 10 “Countdown to 2015” countries on track to meet both MDG4 and MDG5a[[31](#_ENREF_31)]. The “Other LMICs” category varies between 82-132 countries (mean 120), depending on the availability of data. P-values (in parentheses next to indicator name) were calculated by N-way ANOVA. Data scales were altered by factors of 10 to allow all to be measured in approximately the same order of magnitude. *P < .05, **P< .01.

**Figure A. Changes in child health determinants between 1990 and 2010**


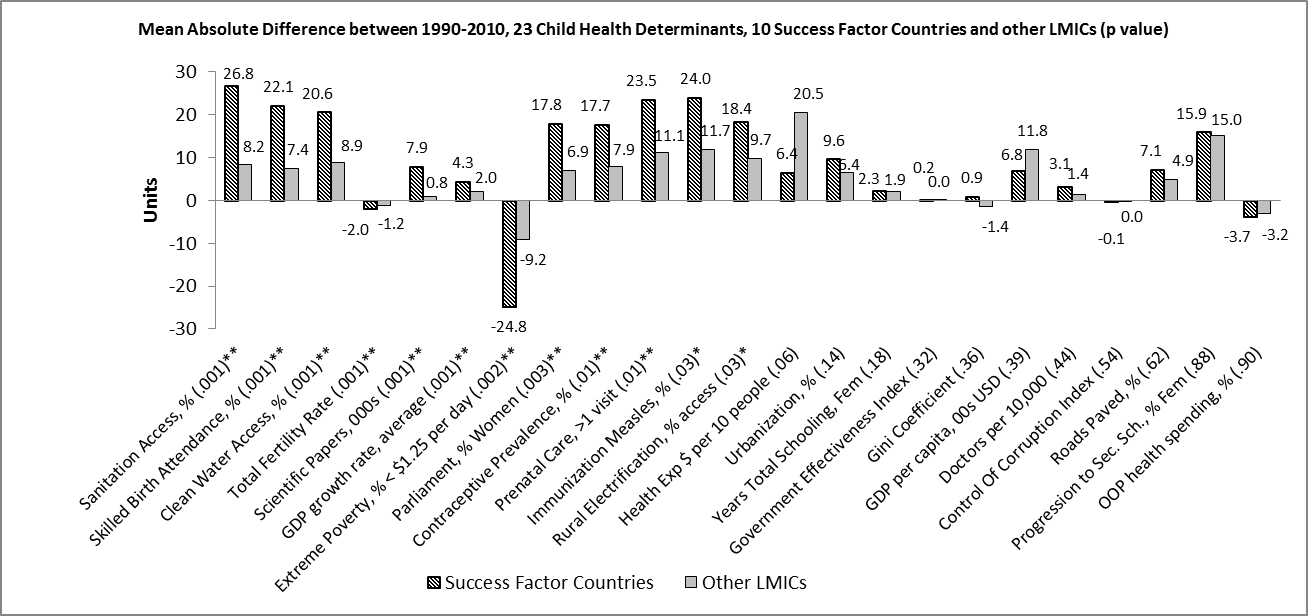


Figure B. Uncertainty ranges shown using box and whisker plots around the effect size of each health determinant (Imputed models).

# Text A STATA CODE

//U5MR loops

global gdpvars "loggdp loggdpppp"

global fertvars "loglag5TFR"

global envvars "logwaterodds logsanitodds"

global educvars "lag5secschoolfem lag10prischoolfem lag5secschool"

global healthsysvars "logoddsSBA logdocs1k"

global immvars "logoddsMeas logoddsDPT"

global womenvars "femlabor womenparl"

global infravars "ruralelec urb"

foreach gdpvar of varlist $gdpvars {

foreach healthsysvar of varlist $healthsysvars {

foreach immvar of varlist $immvars {

foreach fertvar of varlist $fertvars { //Also run without this loop to remove fertility vars

foreach envvar of varlist $envvars {

foreach educvar of varlist $educvars {

foreach womenvar of varlist $womenvars {

foreach infravar of varlist $infravars {

oaxaca logU5mavg `gdpvar' `healthsysvar' `fertvar' `educvar' `womenvar' `immvar’ `infravar’ `envvar’/// [aweight = popavg], by(group) detail

outreg2 using U5MR.xlm, append bdec(3) bracket excel label stats(coef) noaster

}}}}}}}}

//MMR loops

global gdpvars "loggdp loggdpppp"

global fertvars "logTFR logadolfert"

global educvars " lag5secschoolfem lag10prischoolfem lag5secschool "

global healthsysvars " logoddsSBA logdocs1k logoddsprenatal"

global womenvars "femlabor womenparl"

global infravars "ruralelec urb logroaddens"

foreach gdpvar of varlist $gdpvars {

foreach healthsysvar of varlist $healthsysvars {

foreach fertvar of varlist $fertvars {

foreach educvar of varlist $educvars {

foreach womenvar of varlist $womenvars {

foreach infravar of varlist $infravars {

oaxaca logMMR `gdpvar' `healthsysvar' `fertvar' `educvar' `womenvar' `infravar’ [aweight = popavg],/// by(group) detail

outreg2 using MMR.xlm, append bdec(3) bracket excel label stats(coef) noaster

}}}}}}

# BIBLIOGRAPHY

1. Institute for Health Metrics and Evaluation (IHME) (2013) IHME - Accelarating global health progress through sound measurement and accountable science. Seattle, WA.

2. World Health Organization (WHO) (2013) Global Health Expenditure Database: National Health Accounts (NHA).

3. United Nations (UN) Statistics Division (2013) UN Data: A World of Information.

4. United Nations (UN) (2013) International Human Development Indicators.

5. Childinfo United Nations Children's Fund (UNICEF) (2013) Monitoring the Situation of Children and Women. pp. Data on the situation of children and women is collected through the Multiple Indicator Cluster Survey (MICS) programme.

6. World Bank Group (2013) World Bank Data: World Development Indicators.

7. Measure Demographic and Health Surveys (DHS), USAID From the American People (2013) STAT Compiler Building Tables with DHS Data.

8. The World Bank (2001) World Development Report 2002: Building Institutions for Markets. USA World Bank; Oxford University Press

9. United Nations (UN) Statistics Division (2012) Composition of Macro Geographical (Continental) Regions, Geographical Sub-Regions, and Selected Economic and Other Groupings

10. UNICEF, WHO, World Bank, UN DESA Population Division. UN Inter-agency Group for Child Mortality estimation. www.childmortality.org

11. Rajaratnam JK, Marcus JR, Flaxman AD, Wang H, Levin-Rector A, et al. (2010) Neonatal, postneonatal, childhood, and under-5 mortality for 187 countries, 1970-2010: a systematic analysis of progress towards Millennium Development Goal 4. The Lancet 375: 1988-2008.

12. WHO, UNICEF, UNFPA, The World Bank, and the United Nations Population Division. Trends in Maternal Mortality: 1990 to 2013. Geneva, World Health Organization, 2014

13. Sachs J (2005) Investing in Development: A Practical Plan to Achieve the Millennium Development Goals. New York: United Nations Development Programme.

14. World Health Organization (WHO) (2007) Everybody Business: Strengthening Health Systems to Improve Health Outcomes: Who’s Framework for Action. Geneva, Switzerland.
